# Outcomes of visceral leishmaniasis in pregnancy: A retrospective cohort study from South Sudan

**DOI:** 10.1371/journal.pntd.0007992

**Published:** 2020-01-24

**Authors:** Judith E. Pekelharing, Francis Gatluak, Tim Harrison, Fernando Maldonado, M. Ruby Siddiqui, Koert Ritmeijer

**Affiliations:** 1 Médecins Sans Frontières, Amsterdam, Netherlands; 2 Médecins Sans Frontières, Lankien, South Sudan; 3 Royal Tropical Institute, Amsterdam, Netherlands; 4 Médecins Sans Frontières, London, United Kingdom; Hospital Infantil de Mexico Federico Gomez, UNITED STATES

## Abstract

**Introduction:**

Visceral leishmaniasis (VL) is endemic in South Sudan, where outbreaks occur frequently. Because of changes in the immune system during pregnancy, pregnant women are considered particularly vulnerable for developing complications of VL disease, including opportunistic infections. There is limited evidence available about clinical aspects and treatment outcomes of VL in pregnancy. We describe characteristics, maternal and pregnancy outcomes from a cohort of pregnant women with VL.

**Methods:**

We conducted a retrospective analysis using routine programme data from a MSF health facility in Lankien, Jonglei State, South Sudan, between Oct 2014 and April 2018. Records were extracted of women diagnosed with VL while pregnant, and those symptomatic during pregnancy but diagnosed during the first two weeks postpartum. Records were matched with a random sample of non-pregnant women of reproductive age (15–45 years) with VL from the same period.

**Results:**

We included 113 women with VL in pregnancy, and 223 non-pregnant women with VL. Women with VL in pregnancy presented with more severe anaemia, were more likely to need blood transfusion (OR 9.3; 95%CI 2.5–34.2) and were more often prescribed antibiotics (OR 6.0; 95%CI 3.4–10.6), as compared to non-pregnant women with VL. Adverse pregnancy outcomes, including miscarriage and premature delivery, were reported in 20% (16/81) where VL was diagnosed in pregnancy, and 50% 13/26) where VL was diagnosed postpartum. Postpartum haemorrhage was common. Pregnant women were more likely to require extension of treatment to achieve cure (OR 10.0; 95%CI 4.8–20.9), as compared to non-pregnant women with VL. Nevertheless, overall initial cure rates were high (96.5%) and mortality was low (1.8%) in this cohort of pregnant women with VL.

**Conclusion:**

This is the largest cohort in the literature of VL in pregnancy. Our data suggest that good maternal survival rates are possible in resource-limited settings, despite the high incidence of complications.

## Author summary

Visceral leishmaniases (VL), also known as ‘Kala azar’, is a parasitic disease transmitted by sand flies. South Sudan is one of the countries in the world with the highest disease burden of VL. Existing evidence about treatment and outcomes of VL in pregnancy is limited. We described outcomes of pregnant women with VL and women diagnosed with VL in the first two weeks postpartum. Pregnant women with VL were treated with liposomal amphotericin B (AmBisome) in this cohort. Survival rates and initial cure rates of women with VL in pregnancy treated in this resource constrained setting were high. However, these patients often required extension of treatment to achieve cure after the initial standard dose of 30mg/kg AmBisome in six doses. Careful evaluation of patients is needed at the end of the standard treatment regimen, in order to ensure proper (parasitological) cure before discharge. We reported a high incidence of complications (including severe anaemia with a need for blood transfusion), adverse pregnancy outcomes (including miscarriage and premature delivery), and obstetric complications. This research adds to the existing body of knowledge concerning VL in pregnancy, although several questions about this condition remain unanswered. Topics for future research include the immunological response to leishmania infection in pregnancy, and maternal, pregnancy and neonatal outcomes after discharge from VL treatment.

## Introduction

Visceral Leishmaniasis (VL), also known as Kala Azar, is endemic in South Sudan, where it is caused by the protozoan *Leishmania donovani* parasite, and transmitted by phlebotomine sand-flies. [1,2].

South Sudan is among the countries with the highest VL burden worldwide, and outbreaks occur regularly, often fueled by civil unrest and mass displacement [3,4].

VL affects the lymphatic and reticuloendothelial system, and patients typically present with fever, malaise, weight loss and anorexia. Clinical signs are splenomegaly, hepatomegaly, wasting and lymphadenopathy, and the disease causes anaemia and sometimes pancytopenia. Complications occur from anaemia, bleeding, malnutrition and intercurrent infections. Without treatment, the disease can progress rapidly in weeks or months and is typically fatal[2,5].

The current first-line treatment in East Africa for uncomplicated VL outside pregnancy is a 17-day course with a combination therapy of intramuscular injections of a pentavalent antimonial sodiumstibogluconate (SSG) and aminoglycoside paromomycin (PM)[2]. Patients with a high risk of (fatal) toxicity or intolerability to pentavalent antimonials are treated with intravenous infusions of liposomal amphotericin B (AmBisome), as this is much better tolerated. Indications for AmBisome treatment include pregnancy, extreme ages, HIV-coinfection and severe VL. Initial cure rates of 93–97% [2,6] and mortality of 2.8%-3% have been reported in large patient cohorts treated in South Sudan between 2009 and 2015 (with SSG/PM as first-line treatment and AmBisome for specific indications)[7,8].

Pregnant women are a particular patient group, considering the modulated maternal immunological system during pregnancy. Pregnancy is described as a pro-inflammatory and anti-inflammatory condition, depending upon the stage of gestation[9]. Current insights on how the immune system interacts with leishmania infection in pregnancy are based on animal studies. T-cell balances and different resulting cytokine patterns are suggested to explain different scenarios of the maternal response to leishmania infection. For example the Th1-predominant inflammatory immune response is associated with both rejection of pregnancy and control of intracellular Leishmania infections, while a Th2-predominant response is associated with preservation of pregnancy and poor Leishmania infection control[10]. No studies are available looking at the particular susceptibility for pregnant women to develop clinical VL disease after infection.

Treatment of VL in pregnancy is indicated, because the threat of a fatal outcome for the mother, the fetus or the newborn due to untreated VL is much greater than the risk related to adverse drug effects[2]. Several clinical studies and case series on VL in pregnancy are available, but all with relatively small patient numbers. Vertical transmission and adverse pregnancy outcomes have been reported, but the prevalence of congenital VL after maternal disease during pregnancy is not known[11,12]. High rates of miscarriages are reported in pregnant women treatment with SSG, up to 57–69% in some studies[13,14]. Survival rates for pregnant women treated for VL vary; some studies report cure rates as high as 100%[13,14] but case fatality rates of 18% are reported in other settings[15]. AmBisome is now considered the safest therapeutic option for VL in pregnancy and it is the recommended treatment for this group in South Sudan[2,16].

The aim of this study is to describe maternal and perinatal outcomes of patients with VL in pregnancy.

## Methods

A retrospective analysis was conducted, using routinely collected data of a VL cohort from a treatment facility in Lankien, Jonglei state, in the north eastern part of South Sudan. The hospital facility has been run by Médecins Sans Frontières (MSF) since 1993, and provides primary outpatient and inpatient health care services, comprehensive maternity care, and treatment for TB, HIV, and tropical diseases including VL. The population in the catchment area is a pastoralist population who practice cattle husbandry and agriculture. The facility is the only hospital in a large region, and located in an area with high VL endemicity, however, access to the health services is often difficult due to poor road conditions, flooding during the rainy season and insecurity. Between 1,000 and 4,600 VL patients per year have been treated in this facility over the past 10 years. In 2017 almost 50% of all reported VL cases in South Sudan were treated in Lankien.

### Diagnosis and treatment

The following diagnostic procedure was pursued for all patients regardless of their pregnancy status. Patients meeting the clinical case definition (history of fever more than 2 weeks and splenomegaly and/or lymphadenopathy and/or wasting (BMI <16 kg/m^2^ for ages >18 years or weight-for-height <-2 Z score for ages ≤18 years)) underwent further laboratory evaluation to confirm VL following a diagnostic algorithm. Diagnostic tests included the rK39 rapid diagnostic test (IT-Leish, Bio-Rad laboratories, USA) for patients with no prior VL treatment history (suspect primary VL), with a positive result confirming VL. Those testing negative were screened with the leishmania direct agglutination test (DAT, Royal Tropical Institute, Amsterdam, The Netherlands) and a high titer (≥1:6,400) confirmed VL. Those with an intermediate DAT titer (1:800–1:3,200) underwent tissue aspiration (spleen or lymph node) for microscopy. Demonstration of leishmania amastigotes confirmed VL. Tissue aspirate microscopy was used as the initial diagnostic test for all patients with a prior history of VL treatment (suspected VL relapse). A clinical diagnosis of relapse VL was made in patients with a contra-indication for spleen aspiration and negative lymph node results, if there was persistent clinical suspicion for VL and no likely alternative diagnosis. All confirmed VL patients were routinely tested for HIV and malaria with rapid diagnostic tests. The protocol indicated a urine pregnancy test for confirmed female VL patients of reproductive age before start of treatment. Patients were evaluated for possible tuberculosis co-infection based on clinical signs and symptoms and the response to VL treatment. Tuberculosis co-infection was confirmed by sputum microscopy or GeneXpert (cartridge-based nucleic acid amplification test) analysis.

Non-pregnant women with uncomplicated VL were treated with the combination of SSG (20 mg/kg) and PM (15 mg/kg) given on an ambulatory basis over 17 days with daily intramuscular injections. All pregnant women diagnosed with VL and non-pregnant patients with severe VL (according to the MSF treatment protocol) were treated with AmBisome by 6 IV infusions of 5 mg/kg on alternate days.

All patients received nutritional support in the form of therapeutic food, and treatment for dehydration and secondary infections if indicated. The indication for treatment with (intravenous) antibiotics was based on clinical suspicion of a bacterial infection requiring treatment with antibiotics, as diagnostic facilities in the treatment setting were limited. Blood transfusion was considered in case of haemoglobin (Hb) <5.0g/dL, or <7.0g/dL if there were signs of decompensation.

### Study design and data source

Women who were pregnant at time of VL diagnosis were identified from the routine electronic database of VL patients (group 1). A second study group included patients who were already symptomatic during pregnancy but diagnosed with VL within the first two weeks after spontaneous abortion or delivery (either home delivery or hospital delivery). This group therefore includes patients with VL in pregnancy, but diagnosed postpartum (group 2). These patients were identified by screening maternity discharge files. Finally, from the VL database, a random selection of non-pregnant VL-positive women of reproductive age (between 15 and 45 years) were selected to serve as comparison group (group 3) with a ratio of 1:2 for pregnant: non-pregnant.

Patient medical records were reviewed to verify data from the database and to complete missing data. Patient characteristics, complications during treatment and treatment outcomes were compared between the different groups.

### Inclusion and exclusion criteria

Patients discharged from VL treatment between October 2014 and July 2016 and in the period March-April 2018 were considered for analysis. No medical records were available for the period September 2016 until February 2018, as these files had been accidentally destroyed. Records with no information on pregnancy status were excluded. Women of reproductive age, with either a negative urine pregnancy test or a note on the admission card indicating that the patient was not pregnant, were considered to be not pregnant.

### Data collection

Data was transferred into a purpose-made database created using Epidata software (version 4.4.2.0). Key dates, demographic, anthropometric, diagnostic and clinical characteristics of patients, treatment regime, complications during treatment and outcome were recorded.

### Outcome

All patients were evaluated for cure (either by clinical improvement or parasitological cure) after completing the standard treatment regimen. ‘Clinical cure’ was by judgement of the clinician, based on the following signs of cure: absence of fever, regression of spleen, weight gain, increase in Hb and improved appetite. Parasitological cure means that a test-of-cure (ToC) by aspirate microscopy was performed at day 21–28 after initiating treatment to confirm cure at the end of treatment. According to the protocol, a ToC was indicated for patients with an increased risk of treatment failure or relapse (i.e. patients with a prior episode of VL, or patients with inadequate or doubtful clinical response, or patients with a known immune-suppressive condition (HIV or TB co-infection), and pregnant patients up to the first 6 months postpartum).

Treatment was extended if no initial cure was achieved after the standard dose treatment. This is presented as ‘need for extended treatment’. The decision to extend treatment could either be based on a positive ToC, or no/inadequate clinical response. If the treatment was extended based on a positive ToC, the ToC was repeated every week until negative. If treatment was extended based on clinical assessment, a ToC was done to assess the effect of extended treatment.

Treatment outcomes were defined as ‘initial cure at discharge’, ‘defaulter’ or ‘death’. There was no systematic follow up after discharge, because patients returned to areas remote from the treatment centre, and therefore we have limited data about definite cure at six months post-treatment. A defaulter was defined as a patient who did not complete the prescribed treatment, left against medical advice, and had an unknown outcome.

The following pregnancy and neonatal outcomes were recorded: uncomplicated pregnancy outcome (defined as a live term delivery during treatment, or when the patient was still pregnant at time of discharge); adverse pregnancy outcome (defined as first trimester abortion, immature or premature delivery <37 weeks’ gestation, or stillbirth); birth weight. Postpartum haemorrhage and surgical evacuation for retained products of conception were recorded as obstetric/postpartum complications.

For each pregnant women gestation of pregnancy was recorded using three categories: first trimester (<13 weeks’ gestation), second trimester (13–27 weeks’ gestation) and third trimester (>27 weeks’ gestation). Often the exact gestation was not known, but an estimation was made based on history of last menstruation and assessment of the abdomen on admission and/or assessment of the baby postpartum.

### Statistical analysis

Categorical variables are presented using absolute numbers and percentages. Continuous variables are summarised using medians (for non-normally distributed data) and ranges. Where appropriate, differences in proportions were measured using Fisher’s exact test and Pearson χ2 test and the results expressed as odds ratios with 95% confidence intervals. Differences in continuous variables were assessed using non-parametric tests (Mann Whitney U test) as the data were non-normally distributed. Data analysis was conducted using SPSS Statistics (version 25).

### Ethics

This research fulfilled the exemption criteria set by the MSF Ethical Review Board (ERB) for a *posteriori* analyses of routinely collected clinical data, and thus did not require additional clearance from the MSF-ERB. It was conducted with permission from the Medical Director of MSF-Operational Centre Amsterdam. All data were anonymised before analysis.

## Results

**Fig 1:**
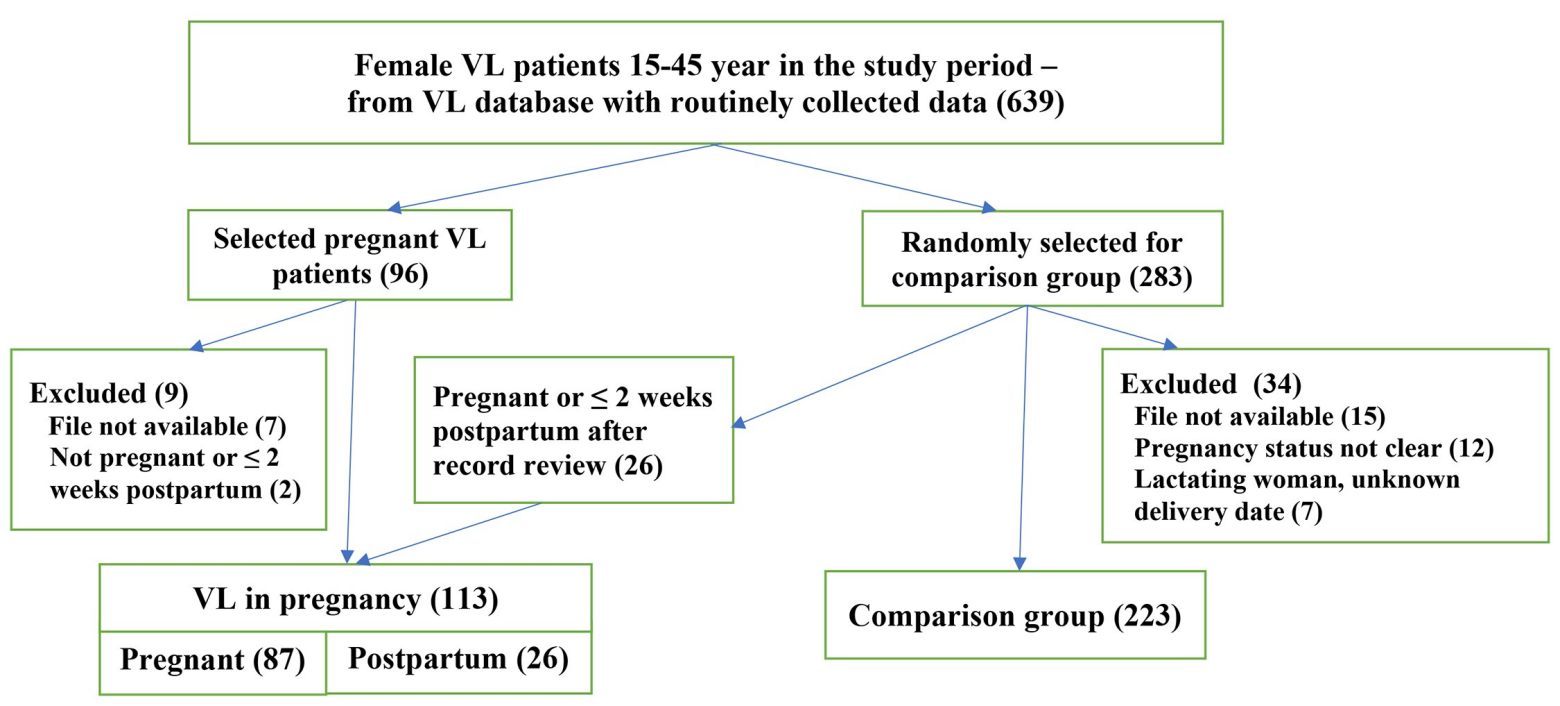
Study flow diagram.

The total cohort of female VL patients in the study period aged 15–45 years was 639. From the database with routinely collected data and by screening maternity discharge files, 96 patients were identified as VL in pregnancy. Seven (7) pregnant patients were excluded because records could not be found. Two additional patients were excluded; one turned out to be non-pregnant, and another one was diagnosed more than 2 weeks post-partum, resulting in 87 pregnant patients with VL included in group 1. Out of the 283 patients that were randomly selected to form the comparison group, 223 were included in group 3 (non-pregnant). Twenty-six (26) were included in group 2 after record review confirming that they had VL diagnosed within the first two weeks postpartum. Thirty-four (34) records were excluded from analysis: 15 because their files could not be found; 12 because the pregnancy status was unclear/not recorded in the file; and 7 were excluded because after record review it turned out they were lactating women with unknown delivery date. See Fig 1 for an overview of the study flow diagram.

### Patient characteristic

Patient characteristics on admission are presented in Table 1.

**Table 1:** Patient characteristics on admission.

	VL in pregnancy		Comparison group	VL in pregnancy (group 1 and 2 combined) compared to Comparison group
	N = 113		N = 223	
	Group 1	Group 2	Group 3	
	Pregnant	Postpartum	Non-pregnant	
	N = 87	N = 26	N = 223	
Age in years	Median 24, range 14–40, IQR 20–29		Median 22, range 15–45, IQR 16–31	p 0.446
Duration of symptoms				
• Up to 4 weeks	76 (91.6%)	24 (96%)	195 (89.9%)	p 0.543
• > 4 weeks	7 (8.4%)	1 (4.0%)	22 (10.1%)	
Missing data	4	1	6	
Splenomegaly				
• No splenomegaly	17 = 30.9%	8 (30.8%)	63 (28.3%)	p 0.994
• 1–4 cm	22 = 40.0%	6 (23.1%)	90 (40.4%)	
• 5-7cm	11 = 20.0%	8 (30.8%)	44 (19.7%)	
• >7cm	5 = 9.1%	4 (15.4%)	26 (11.7%)	
Missing data *	32			
Nutritional status, BMI in kg/m2**				
• < 13.0	1 = 1.4%	0 = 0%	4 = 2.9%	p < 0.001
• 13.0–14.4	1 = 1.4%	3 = 14.3%	26 = 19.1%	
• 14.5–15.9	9 = 12.9	5 = 23.8%	39 = 28.7%	
• ≥ 16.0	59 = 84.3%	13 = 61.9%	67 = 49.3%	
Hb on admission in g/dL				
• <6.0 g/d	2 = 2.3%	2 = 7.7%	4 = 1.8%	p < 0.001
• 6.0–6.9	10 = 11.5%	4 = 15.4%	7 = 3.1%	
• 7.0–8.9	28 = 32.2%	15 = 57.7%	68 = 30.5%	
• ≥ 9.0	47 = 54.0%	5 = 19.2%	144 = 64.6%	
Malaria	2/75 (2.7%)	2/24 (7.7%)	3/136 (2.2%)	p 1.000
Missing data	12	2	87	OR 1.2 (0.20–7.6)
HIV	0/67 (0%)	0/21 (0%)	0/206 (0%)	p 1.000
Missing data	20	5	17	
Primary VL	99 (87.6%)		196 (87.9%)	p 1.000
Relapse VL	14 (12.4%)		27 (12.1%)	
Diagnosis of primary VL by positive rk39	97.3%	81%	94.9%	p 0.785

* Spleen size data were missing for 32 pregnant women in the third trimester, as spleen exam was not feasible or was unreliable in advanced pregnancy

** presented for patients ≥19 years

Of the patients with VL in pregnancy (group 1 and 2 combined), 26.8% were in the first trimester of pregnancy at the time of diagnosis, 35.1% in the second trimester and 38.1% in the third trimester. Among the patients in the first trimester, four patients had symptoms for more than one month. These patients could possibly have been symptomatic before they became pregnant. The remaining patients (99/113) were pregnant before they became symptomatic with VL. Parity of pregnant VL patients (group 1) was distributed as follows: nullipara: 17.2%, subsequent pregnancies: 82.8% (range para 1–8).

The great majority of patients with primary VL (93%) was diagnosed with a positive rk39 rapid diagnostic test. A small group of primary VL patients required further diagnostic follow up. All VL relapse patients were diagnosed using lymphnode or spleen aspirate microscopy. Thirty percent (30%) of records had no information about malaria status and 12.5% had no information about HIV status.

### Complications during treatment

Recorded complications during treatment are presented in Table 2.

**Table 2:** Complications during VL treatment.

	VL in pregnancy		Comparison group	VL in pregnancy (group 1 and 2 combined) compared to Comparison group
	N = 113		N = 223	
	Group 1	Group 2	Group 3	
	Pregnant	Postpartum	Non-pregnant	
	N = 87	N = 26	N = 223	
Bleeding	13 = 14.9%	7 = 26.9%	11 = 4.9%	p < 0.01
Missing data	9	1	5	OR 4.5 (2.1–9.9)
Blood transfusion	7 (9.7%)	4 (17.4%)	3 (1.4%)	p < 0.001
Missing data	15	3	7	OR 9.3 (2.5–34.1)
Jaundice (on admission or during treatment)	7 (9.5%)	1 (4.0%)	6 (4.2%)	p 0.265, OR 2.0 (0.67–5.9)
Missing data	13	1	81	
Antibiotic treatment for suspected infectious complications				
- Total (iv. or oral)	49 (66.2%)	22 (88%)	40 (29.9%)	p < 0.001
- Intravenous	34 (45.9%)	16 (64%)	10 (7.5%)	OR 6.0 (3.4–10.6)
Missing data	13	1	89	

The most commonly reported bleeding complications were nose-bleeding and obstetric related bleeding. Bleeding after an abortion and postpartum haemorrhage was also recorded as bleeding complication in this table.

Significantly more blood transfusions were reported for patients with VL in pregnancy, compared to the comparison group. Patients who received blood transfusion had a median lowest Hb value of 5.1 g/dL, ranging from 3.5 to 7.3 g/dL.

Antibiotics were prescribed for infectious complications, including suspected sepsis in severely ill patients, respiratory tract infection, ear infection, urinary tract infection, eye infection, skin infection, abscess, and sometimes because of ongoing fever with no identified focus. Some patients diagnosed postpartum were treated with antibiotics for fever postpartum (suspected endometritis) before VL diagnosis.

Two pregnant patients were inadvertently started on SSG/PM treatment because the clinician was unaware of their pregnancy status. Both patients had an adverse pregnancy outcome (first trimester abortion and immature delivery respectively).

Change of treatment regimen from SSG/PM to AmBisome (due to SSG toxicity, jaundice or deteriorating condition) occurred in 7 (3.6%) of non-pregnant patients and none of the patients with VL postpartum.

### Treatment outcomes

Treatment outcomes are presented in Table 3.

**Table 3:** Treatment outcomes.

	VL in pregnancy		Comparison group	VL in pregnancy (group 1 and 2 combined) compared to Comparison group
	N = 113		N = 223	
	Group 1	Group 2	Group 3	
	Pregnant	Postpartum	Non-pregnant	
	N = 87	N = 26	N = 223	
Treatment regimen				
• AmBisome	85 (97.7%)	19 (73.1%)	27 (12.1%)	p < 0.001
• SSG/PM	2 (2.3%)	7 (26.9%)	196 (87.9%)	
Treatment extended because no cure at the end of standard treatment				
- Total	31 (35.6%)	12 (46.1%)	22 (9.8%)	p< 0.001 OR 5.7 (3.2–10.2)
- Based on positive ToC	10 (11.4%)	8 (30.7%)	11 (4.9%)	p< 0.001 OR 4.1 (1.9–8.6)
- Based on no clinical cure	20 (22.9%)	3 (11.5%)	9 (4.0%)	p < 0.001OR 6.0 (2.7–13.4)
Reason not documented	1	1	2	
Total dose AmBisome*				
No. of doses				
- 6	50 (64.9%)	8 (44.4%)	12 (48.0%)	p 0.485
- 7–8	18 (23.4%)	4 (22.3%)	10 (40.0%)	
- 9–10	7 (9.1%)	1 (5.6%)	3 (12.0%)	
- >10	2 (2.6%)	5 (27.8%)	0	
Missing data	5	0	1	
Treatment outcome				
- Initial cure at discharge	84 (96.6%)	25 (96.2%)	218 (97.8%)	p 0.739
- Defaulter	2 (2.3%)	0	3 (1.3%)	
- Death	1 (1.1%)	1 (3.8%)	2 (0.9%)	
Among those cured:				
- Clinical cure	34 (40.5%)	7 (28.0%)	166 (76.1%)	p < 0.001
- Parasitological cure	50 (59.5%)	18 (72.0%)	52 (23.9%)	
Discharge Hb in g/dL				
• <6.0 g/dL	1 = 1.2%	0 = 0%	1 = 0.5%	p < 0.001
• 6.0–6.9	12 = 14.3%	3 = 12.5%	2 = 0.9%	
• 7.0–8.9	41 = 48.8%	16 = 66.7%	37 = 17.1%	
• ≥ 9.0	30 = 35.7%	5 = 20.8%	176 = 81.5%	
missing data	3	2	7	

* For patients who were started on AmBisome as initial treatment regimen and excluding defaulters and deaths. One dose of AmBisome is 5mg/kg.

One pregnant woman with severe VL disease died after the first dose of AmBisome. One patient died after being diagnosed with VL postpartum (associated with suspected sepsis).

For four patients with VL postpartum the treatment regimen was changed to SSG/PM after a full course of AmBisome due to unsatisfactory treatment response.

For the patients in the comparison group, the treatment regimen was changed from SSG/PM to AmBisome in 7 cases (3.6%), but only because of SSG toxicity or deteriorating condition, and not due to treatment failure.

Initial cure rates at time of discharge were not significantly different between the groups. When comparing the patients with VL in pregnancy to the patients in the non-pregnant group who were treated with AmBisome, there was no significant difference in need for treatment extension or the total dose of AmBisome. Looking at all the groups combined, patients with relapse VL had higher odds of requiring extended treatment to achieve cure (OR 2.2, 95% CI 1.05–4.50 p 0.037).

### Obstetric and perinatal outcomes

Data about pregnancy outcomes and obstetric complications from time of VL diagnosis until discharge from VL treatment are presented in Table 4.

**Table 4:** Obstetric and pregnancy outcomes.

	Group 1	Group 2
	Pregnant	Postpartum
	N = 81	N = 26
Still pregnant at time of discharge	64 (79%)	N/A
Delivered during treatment	17 (21%)	
Adverse pregnancy outcome	16 (20%)	13 (54%)
	Among those who delivered	
Post-partum haemorrhage	6/17 (35%)	*
Surgical evacuation for retained products of conception	5/17 (29%)	6/24 (25%)

* No reliable data from home deliveries

Stillbirth occurred in two cases: in a patient who delivered during treatment by caesarean section in the third trimester for bleeding placenta praevia, and in a patient diagnosed postpartum who delivered on the road just before admission (unknown gestation). Insufficient data were available in the patient files to trace other neonatal outcomes such as apgar score or neonatal survival rates after birth.

Pregnancy outcomes per trimester are presented in Table 5, for patients who were pregnant at time of VL diagnosis. Adverse pregnancy outcomes occurred more frequently in the third trimester compared to the second trimester (p 0.001, OR 14.0, 95%CI 2.8–75.6), differences between the other trimesters were not significant. Data about trimester of pregnancy were missing in 15% (13/87) cases.

**Table 5:** Pregnancy outcomes per trimester.

	Uncomplicated pregnancy outcome (pregnant on discharge or live term delivery)	Adverse pregnancy outcome (miscarriage, premature delivery < 37 wks gestation, stillbirth)
Diagnosed with VL during 1^st^ trimester N = 22	17 (77.3%)	5 miscarriages (22.7%)
Diagnosed with VL during 2^nd^ trimester N = 33	31 (93.9%)	2 miscarriages/immature deliveries (6.1%)
Diagnosed with VL	9 pregnant on discharge (47.4%)	9 premature deliveries (47.4%) of
during 3^rd^ trimester: N = 19	1 live term delivery (5.3%)	which 1 stillbirth

Adverse pregnancy outcomes occurred more frequently among primigravidae compared with subsequent pregnancies (OR 4.33, 95% CI 1.03–18.2), although this should be interpreted carefully as data about parity were available for only 66.7% (58/87) of cases.

Twenty-nine patients delivered in the third trimester (during treatment or before starting treatment). Data on birth weights are available for the babies of only 13 patients. The median weight was 1.9kg, range 1.2–2.6kg.

## Discussion

### Patient characteristics

The different patient groups were similar regarding age, duration of symptoms, and proportion of primary VL/relapse VL (no significant difference between the groups). BMI was higher among pregnant women, but this is likely to be due to the pregnancy and not representing a better nutritional status.

Hb on admission was significantly lower for pregnant women compared to non-pregnant women, and lower for postpartum women compared to pregnant women. The difference can be explained by the (physiologic) anaemia in pregnancy and blood loss during delivery.

None of the women in this cohort was diagnosed with HIV infection during routine testing. Missing data are likely due to recording omissions of negative test results. Few patients in this cohort were diagnosed with TB co-infection. TB-incidence in South Sudan was estimated 146/100,000 in 2017[17]. However, because a higher TB incidence can be expected in immune-suppressed VL patients[18], TB may have been underdiagnosed, as diagnosis of (extra-pulmonary) TB is challenging.

Also, although malaria is highly endemic in South Sudan, with 159 infections per 1,000 people at risk in 2016[19], few malaria co-infections were reported. A possible explanation could be that patients were treated for malaria in the out-patient clinic before being tested for VL. This information was not available, as patients take their out-patient medical records home. Rates of malaria-VL coinfection varied between 3.8 and 60.8% in a study from Sudan, depending on the geographical setting[20].

Detailed general population data are lacking to determine whether women in certain trimesters are more susceptible to develop VL after infection and whether susceptibility is influenced by parity.

### Disease related complications

Blood transfusions were needed significantly more often in patients with VL in pregnancy (OR 9.3 (2.5–34.1)). This is something to consider when planning VL treatment services.

Jaundice (either on admission or occurring during treatment) was reported in 8% of pregnant women with VL, and is a sign impaired liver function due to severe VL, and is an indication for treatment with AmBisome also for non-pregnant patients.

Antibiotics were more often prescribed in pregnant and postpartum women compared to non-pregnant patients (OR 6.0 (3.4–10.6)). There may have been over-diagnosis of suspected bacterial infections in patients with ongoing fever after a few days of treatment. On the other hand, VL is an immune-suppressive condition and secondary infections are a known complication. Empiric antibiotic therapy can be a reasonable choice for certain patients in this resource constrained setting. It is possible that physicians were more likely to indicate empiric antibiotic treatment for pregnant women and patients admitted postpartum when an infectious focus was not identified.

### Treatment outcomes

Mortality during treatment in patients with VL in pregnancy (group 1 and 2 combined) was 1.8% (2/133).

This is comparable to large VL cohorts[7,8], but lower than some other reports about VL in pregnancy[15,21]. A Sudanese study reported 18% case fatality rate among 45 pregnant patients treated with SSG in 2014–2015, mainly due to hepatic failure, bleeding manifestation, and severe anaemia/heart failure[15]. In another study from Sudan where pregnant women with VL were treated with SSG, the main cause of dead was hepatic encephalopathy[21]. In our study cohort it is likely that especially the severely ill patients, including the patients with jaundice, have benefited from treatment with AmBisome, and blood-transfusion may have been a live-saving intervention in some cases.

Case fatality rate in all non-pregnant female patients in reproductive age in the cohort was 2.8% during the study period (source: database with routinely collected data), which is comparable to previous reports[7,8].

For the patients with VL in pregnancy, treatment was more often extended compared to the non-pregnant comparison group (OR 5.7 (3.2–10.2)). For some patients treated with AmBisome, the standard treatment of 30mg/kg divided in six doses was extended to up to 12 doses (60mg/kg) to achieve cure. There was no significant difference in mean total dose of AmBisome between patients with VL in pregnancy and patients treated with AmBisome in the comparison group. The patients in the comparison group treated with AmBisome often had severe disease, which could explain why many of them also needed extended treatment. Higher frequency of extended treatment in pregnant women could partly be explained by the fact that pregnant women are routinely tested for parasitological cure, whereas for non-pregnant women this is only done in case of suspected lack of cure by clinical evaluation.

A proportion of patients with VL in pregnancy were discharged with clinical cure, but without parasitological confirmation of cure (which is the protocol for patients with VL in pregnancy). A ToC may not have been possible because of spleen regression, or because of advanced pregnancy as contraindication for splenic aspiration with no lymphadenopathy as alternative aspirate site.

A recent report of treatment outcomes for VL from Ethiopia showed initial cure rates of 96.7% for non HIV-co-infected patients treated with AmBisome (total dose 24-35mg/kg) [22,23]. In our cohort, initial cure was achieved for only 61% of pregnant women treated with the standard dose of AmBisome (30mg/kg), while 35.6% required extension of treatment to achieve initial cure, indicating an impaired treatment response in pregnant women. This is similar to the initial cure rates of 60% for HIV co-infected patients after treatment with 30mg/kg AmBisome[24], and likely due to the modulated immune system during pregnancy. This justifies the routine ToC after the standard dose of AmBisome (of 30mg/kg) for pregnant women with VL to establish proper parasitological cure before discharge.

Many pregnant and postpartum women were discharged anaemic, despite routine treatment with ferrous sulphate and folic acid for all patients. This is a risk especially for pregnant women who may deliver at home after discharge from VL treatment.

### Obstetric and perinatal outcomes

Twenty-one percent (17/81) of pregnant patients delivered during or directly after finishing treatment. Most adverse pregnancy outcomes involved miscarriages in the first trimester or premature deliveries in the third trimester. Although a proportion of the adverse pregnancy outcomes was probably caused by VL, no community-based studies are available to compare these pregnancy outcomes with the general population. It is interesting that only two adverse pregnancy outcomes (6.1%) were recorded for women with VL admitted in the second trimester. The relation between adverse pregnancy outcomes and different immunological states of the pregnant women in different trimesters remains a point for future research, as well as the relation between (different trimesters in) pregnancy and susceptibility for developing VL.

Significantly more adverse pregnancy outcomes were found among primigravidae (OR 4.33, 95% CI 1.03–18.2). A similar outcome is known for malaria infection during pregnancy, with placental malaria with adverse outcomes being more common among primigravidae, although the mechanism may be different. Much about the immunological response and pregnancy outcomes per trimester remains unknown. It would be relevant to consider studying placenta’s of VL patients after delivery for the presence of placental invasion with parasites, including those who experience premature deliveries during treatment or after apparently successful treatment.

When interpreting the perinatal outcomes, it should be noted that exact gestation of pregnancy was often not known, but estimated based on history of last menstruation and assessment of the abdomen on admission and/or assessment of the baby postpartum. No documentation from early-pregnancy antenatal care visits or ultrasound reports were available. A differentiation between intra-uterine growth restriction or low birth weight due to prematurity is therefore difficult.

Data about (home) deliveries after discharge from VL treatment were not available. Because no follow-up data were available, it is possible that the actual number of adverse pregnancy outcomes is higher than the presented figures. Moreover, no follow-up data were available for neonatal outcomes. Data about the condition of the neonate after delivery until discharge were also not available, as the neonates often had their own patient records which we were not able to identify from the archive. Preterm birth is the most important determinant of adverse infant outcomes in terms of survival and quality of life globally, and adverse outcomes are significant especially in low resource settings[25,26].

Data about birth weight and gestation at time of delivery were available for the babies of only 13 patients (out of 43 patients who delivered before or during treatment), therefore no conclusions can be drawn from these numbers. The available data show low birth weight deliveries, which is in line with the high number of premature deliveries.

Data about blood loss during delivery were not available from patients admitted postpartum after home delivery. Post-partum haemorrhage was reported in 35% of in-hospital deliveries, but did not lead to any maternal mortality, probably thanks to the presence of skilled health personnel, and availability of uterotonic drugs and blood transfusion.

Surgical evacuation for retained products of conception (either manual vacuum aspiration or curettage) was reported in 29% of cases. Retained placenta is known to occur more frequently in women with preterm vaginal delivery than in women with term vaginal delivery (9.1% vs 1.1)[27]. In VL patients it may be difficult to distinguish between fever due to VL and infected retained products of conception. In already anaemic patients, prone for infectious complications and bleeding, the threshold for clinicians to perform evacuation may have been lower than in non-VL patients. A high rate of retained products of conception in VL patients has not been reported elsewhere.

### Strengths and limitations

We described a large cohort of patients diagnosed with VL in pregnancy and the early postpartum period. Previous publications about VL in pregnancy are limited in number and have smaller patient cohorts. Availability of patient records in general was good and data about patient characteristics on admission and treatment outcomes were complete in most cases.

The major limitation of the study is missing data for some of the outcomes, especially for pregnancy and neonatal outcomes, due to the retrospective study design. Pregnancy outcomes after discharge from VL treatment and neonatal outcomes after birth remain an important point for future research, ideally with prospective studies. The same accounts for final VL outcomes, as the incidence of relapse VL after initial successful treatment of VL in pregnancy is not known. However, longitudinal follow-up is very challenging in this setting in South Sudan, where patients return to their (often remote) villages after completing treatment, with important access barriers due to poor infrastructure and public transport, especially during the rainy season, and general insecurity.

This study demonstrates that in order to achieve favorable treatment outcomes a certain level of care is needed for the treatment of VL in pregnancy, with the ability to manage potential complications of the disease. We mentioned the need for treatment with AmBisome (which requires cold chain and intravenous administration), intravenous antibiotics, blood transfusion, management of obstetric complications and care for premature neonates. These services are available in the MSF health facility in Lankien in the current set-up, but may be very challenging to realize in all VL treatment sites in South Sudan, as they are typically in remote and extremely resource-limited locations with severe capacity and logistic constraints.

### Conclusion

This is the largest cohort reporting patient characteristics and maternal and pregnancy outcomes for patients with VL in pregnancy treated with AmBisome. We reported high survival rates and cure rates at discharge for women with VL in pregnancy, treated in a low resource setting. However, patients with VL in pregnancy treated with AmBisome often required extension of treatment to achieve cure after the initial total standard dose of 30mg/kg. Careful evaluation of patients is needed at the end of the standard treatment regimen, in order to ensure proper (parasitological) cure before discharge.

Follow up data would be relevant to determine if women with VL in pregnancy have an increased risk for relapse of VL disease, and to understand the risk of pregnancy-related complications after discharge. Commonly reported complications, such as suspected secondary infections, severe anaemia and obstetric complications, emphasize the need for appropriate medical care in addition to VL treatment for this patient group.

## Supporting information

S1 Checklist STROBE checklist. (DOC)
