# Supplementary material for: Outcomes of visceral leishmaniasis in pregnancy: A retrospective cohort study from South Sudan
Source: PLoS Negl Trop Dis. 2020 Jan 24;14(1):e0007992. doi: 10.1371/journal.pntd.0007992 (PMC7001985; doi:10.1371/journal.pntd.0007992)
Supplement: S1 Checklist — (DOC) [file pntd.0007992.s001.doc]

STROBE Statement—Checklist of items that should be included in reports of ***cohort studies***

|  | Item No | Recommendation | Manuscript VL in pregnancy |
| --- | --- | --- | --- |
| **Title and abstract** | 1 | (*a*) Indicate the study’s design with a commonly used term in the title or the abstract | Title page, title |
|  |  | (*b*) Provide in the abstract an informative and balanced summary of what was done and what was found | Abstract and authors summary |
| Introduction | | |  |
| Background/rationale | 2 | Explain the scientific background and rationale for the investigation being reported | Introduction, paragraph 1-4. |
| Objectives | 3 | State specific objectives, including any prespecified hypotheses | Introduction, last sentence= line 97. |
| Methods | | |  |
| Study design | 4 | Present key elements of study design early in the paper | Methods, section ‘Study design and data source’ |
| Setting | 5 | Describe the setting, locations, and relevant dates, including periods of recruitment, exposure, follow-up, and data collection | Methods, first paragraph and section ‘Inclusion and exclusion criteria’ |
| Participants | 6 | (*a*) Give the eligibility criteria, and the sources and methods of selection of participants. Describe methods of follow-up | Methods, section ‘Study design and data source’ |
|  |  | (*b*) For matched studies, give matching criteria and number of exposed and unexposed | N/A |
| Variables | 7 | Clearly define all outcomes, exposures, predictors, potential confounders, and effect modifiers. Give diagnostic criteria, if applicable | Methods, section ‘Diagnosis and treatment’ for diagnostic criteria and ‘Outcome’ for outcome variables |
| Data sources/ measurement | 8* | For each variable of interest, give sources of data and details of methods of assessment (measurement). Describe comparability of assessment methods if there is more than one group | Methods, section ‘Study design and data source’ |
| Bias | 9 | Describe any efforts to address potential sources of bias | Discussion, section ‘Treatment complications’ (paragraph 2) and section ‘Strengths and limitations’ |
| Study size | 10 | Explain how the study size was arrived at | Methods, section ‘Study design and data source’ |
| Quantitative variables | 11 | Explain how quantitative variables were handled in the analyses. If applicable, describe which groupings were chosen and why | Methods, section ‘Statistical analysis’ |
| Statistical methods | 12 | (*a*) Describe all statistical methods, including those used to control for confounding | Methods, section ‘Statistical analysis’ |
|  |  | (*b*) Describe any methods used to examine subgroups and interactions | Methods, section ‘Statistical analysis’ |
|  |  | (*c*) Explain how missing data were addressed | Missing data are reported in the results section. |
|  |  | (*d*) If applicable, explain how loss to follow-up was addressed | N/A |
|  |  | (*e*) Describe any sensitivity analyses | N/A. P values are provided for comparisons between groups. |
| Results | | |  |
| Participants | 13* | (a) Report numbers of individuals at each stage of study—eg numbers potentially eligible, examined for eligibility, confirmed eligible, included in the study, completing follow-up, and analysed | Results, paragraph 1 |
|  |  | (b) Give reasons for non-participation at each stage | Results, paragraph 1 |
|  |  | (c) Consider use of a flow diagram | Results, figure 1 |
| Descriptive data | 14* | (a) Give characteristics of study participants (eg demographic, clinical, social) and information on exposures and potential confounders | Results, section ‘Patient characteristics’ paragraph 1-2 |
|  |  | (b) Indicate number of participants with missing data for each variable of interest | In all tables in results section. |
|  |  | (c) Summarise follow-up time (eg, average and total amount) | Methods, section ‘Outcome’. |
| Outcome data | 15* | Report numbers of outcome events or summary measures over time | Results, section ‘Complications during treatment, ‘Treatment outcomes’, ‘Obstetric and perinatal complications’ |
| Main results | 16 | (*a*) Give unadjusted estimates and, if applicable, confounder-adjusted estimates and their precision (eg, 95% confidence interval). Make clear which confounders were adjusted for and why they were included | Results, section ‘Complications during treatment, ‘Treatment outcomes’, ‘Obstetric and perinatal complications’ |
|  |  | (*b*) Report category boundaries when continuous variables were categorized | Tables 2-4 in Results section |
|  |  | (*c*) If relevant, consider translating estimates of relative risk into absolute risk for a meaningful time period | Tables 2-4 in Results section |
| Other analyses | 17 | Report other analyses done—eg analyses of subgroups and interactions, and sensitivity analyses | Results section. |
| Discussion | | |  |
| Key results | 18 | Summarise key results with reference to study objectives | Discussion, last paragraph |
| Limitations | 19 | Discuss limitations of the study, taking into account sources of potential bias or imprecision. Discuss both direction and magnitude of any potential bias | Discussion, section ‘Strengths and limitations’ |
| Interpretation | 20 | Give a cautious overall interpretation of results considering objectives, limitations, multiplicity of analyses, results from similar studies, and other relevant evidence | Discussion, section ‘Treatment outcomes’, paragraph 3.  Discussion, section ‘Obstetric and perinatal outcomes’, paragraph 2  Conslusion |
| Generalisability | 21 | Discuss the generalisability (external validity) of the study results | Discussion, section ‘Strengths and limitaions’, paragraph 3 |
| Other information | | |  |
| Funding | 22 | Give the source of funding and the role of the funders for the present study and, if applicable, for the original study on which the present article is based | See Funding statement section in submission. |

*Give information separately for exposed and unexposed groups.

**Note:** An Explanation and Elaboration article discusses each checklist item and gives methodological background and published examples of transparent reporting. The STROBE checklist is best used in conjunction with this article (freely available on the Web sites of PLoS Medicine at http://www.plosmedicine.org/, Annals of Internal Medicine at http://www.annals.org/, and Epidemiology at http://www.epidem.com/). Information on the STROBE Initiative is available at http://www.strobe-statement.org.
